# Clinical performance of the fully automated Lumipulse plasma p‐tau217 assay in mild cognitive impairment and mild dementia

**DOI:** 10.1002/dad2.70080

**Published:** 2025-02-14

**Authors:** Adam H. Dyer, Jean Dunne, Helena Dolphin, Laura Morrison, Antoinette O'Connor, Sarah Fullam, Tara Kenny, Aoife Fallon, Sean O'Dowd, Nollaig M. Bourke, Niall P. Conlon, Sean P. Kennelly, for the TIMC‐BRAiN Study Group

**Affiliations:** ^1^ Tallaght Institute of Memory and Cognition Tallaght University Hospital Dublin Ireland; ^2^ Discipline of Medical Gerontology School of Medicine Trinity College Dublin Dublin Ireland; ^3^ Trinity Translational Medicine Institute Trinity College Dublin Dublin Ireland; ^4^ Clinical Immunology Laboratory St James's Hospital Dublin Ireland; ^5^ Department of Neurology Tallaght University Hospital Dublin Ireland; ^6^ Academic Unity of Neurology School of Medicine Trinity College Dublin Dublin Ireland; ^7^ Clinical Medicine School of Medicine Trinity College Dublin Dublin Ireland

**Keywords:** Alzheimer's disease, amyloid, biomarker, cerebrospinal fluid, plasma, tau

## Abstract

**Introduction:**

Plasma phosphorylated tau (p‐tau)217 is a leading blood‐biomarker for the detection of amyloid beta (Aβ) pathology. We assessed the performance of a fully automated plasma p‐tau217 immunoassay to detect Aβ pathology in mild cognitive impairment (MCI)/mild dementia.

**Methods:**

Paired plasma and cerebrospinal fluid (CSF) samples were obtained at time of diagnostic lumbar puncture (LP) in a specialist memory service. Plasma p‐tau217 was measured using the Lumipulse immunoassay platform and ability to detect CSF‐defined Aβ positivity assessed.

**Results:**

Of 148 participants (69.4 ± 6.5 years; 54.1% female), 101 had MCI and 47 mild dementia. Median plasma p‐tau217 was > 4‐fold higher in Aβ+ vs Aβ− individuals with an area under the curve of 0.92 (0.87–0.97). Application of 90%, 95%, and 97.5% sensitivity/specificity thresholds for plasma p‐tau217 may have obviated the need for more than half of LPs.

**Discussion:**

Our real‐world data support the clinical use of fully automated plasma p‐tau217 immunoassays, although further studies in more diverse cohorts are required.

**HIGHLIGHTS:**

- Plasma phosphorylated tau (p‐tau)217 was measured using a fully automated immunoassay (Lumipulse).
- P‐tau217 was > 4‐fold higher in amyloid beta (Aβ)+ versus Aβ− individuals.
- Plasma p‐tau217 had an area under the curve of 0.92 for detection of Aβ status.
- Using a previously proposed two‐threshold approach may avoid more than half of lumbar punctures.

## 1 BACKGROUND

The development of cerebrospinal fluid (CSF) and positron emission tomography (PET) markers of amyloid beta (Aβ) and tau (T) pathology has enabled clinico‐biological diagnosis of Alzheimer's disease (AD).^1^, ^2^, ^3^ Establishing Aβ positivity is increasingly important with anti‐amyloid immunotherapies potentially demonstrating benefit in AD.^4^ Blood‐based biomarkers (BBMs) have clear advantages over CSF/PET and are set to transform pathways for diagnosis, trial recruitment, and disease monitoring in AD.^5^ Among BBMs, phosphorylated tau (p‐tau)217 may differentiate AD from other neurodegenerative diseases, exhibits a large fold change, and tracks worsening cortical atrophy over time.^6^, ^7^, ^8^

Although mass spectrometry is considered the gold standard for quantifying low‐abundant plasma proteins such as p‐tau217, newer immunoassay technologies can be used to measure plasma p‐tau217 and demonstrate excellent performance in detecting Aβ pathology.^9^ These include partially automated (e.g., Simoa^10^) and fully automated methods (e.g., Fujirebio Lumipulse), which may exhibit similar performance to Simoa.^11^, ^12^, ^13^ Fully automated workflows may reduce variability in assay performance over time and use technology already in place in many diagnostic laboratories.

Further studies examining the performance of plasma p‐tau217 in “real‐world” clinical cohorts are needed. This includes specialist memory services as assay performance is inevitably tied to context/cohort under study.^14^ We assessed the performance of the fully automated Fujirebio Lumipulse G plasma p‐tau217 assay to detect CSF‐defined Aβ pathology in individuals undergoing lumbar puncture (LP) as part of diagnostic work‐up in a regional specialist memory clinic (RSMC).

## 2 METHODS

### 2.1 Study participants

Data were included from 148 consecutive participants who donated paired plasma and CSF samples to the Tallaght Institute of Memory and Cognition Biobank for Research in Aging and Neurodegeneration (TIMC‐BRAiN).^15^ The RSMC assesses 400 to 500 individuals annually, including ≈ 200 new referrals who undergo detailed cognitive/medical assessment, neuroimaging, and routine laboratory investigations. If appropriate, CSF testing to detect AD pathology is offered in mild cognitive impairment (MCI)/mild dementia and is conducted in approximately two fifths of new cases attending this RSMC.^16^

For TIMC‐BRAiN participants, each diagnosis was discussed at a dedicated consultant‐led biobank meeting. Diagnosis and stage were adjudicated based on functional status (MCI/dementia) and a suspected etiological diagnosis (AD, Lewy body disease, frontotemporal dementia, etc.) incorporating review of LP results.^15^ TIMC‐BRAiN has received ethical approval from the Tallaght‐St. James's Research Ethics Committee (Project ID: 2159).

### 2.2 Sample collection, processing. and analysis

Plasma samples were obtained at time of LP alongside CSF samples. Plasma samples were drawn in K_2_EDTA Vacuettes, centrifuged (1800 g × 10 minutes) and immediately stored in sterile cryovials at −80°C. CSF was centrifuged immediately (400 g × 10 minutes) and stored in sterile polypropylene cryovials at −80°C. CSF was batch analyzed for Aβ40, Aβ42, and p‐tau181 using in vitro diagnostic (IVD) CSF Kits on a Lumipulse G II 600 analyzer. A CSF Aβ42:Aβ40 ratio of ≤ 0.067 was considered indicative of Aβ pathology (A+)^17^ and CSF p‐tau181 of ≥ 51.6 pg/mL indicative aberrant tau phosphorylation (T+).^17^

**Research in context**

1. **Systematic review**: Traditional (e.g., PubMed), preprint servers and conference presentations were reviewed. There is clear evidence that plasma phosphorylated tau (p‐tau)217 can detect amyloid beta (Aβ) pathology characteristic of Alzheimer's disease. Several immunoassay platforms measuring p‐tau217 demonstrate excellent performance in cohort studies; however, fewer studies have been conducted in memory clinic contexts.
2.  **Interpretation**: In a specialist memory service, the fully automated Lumipulse plasma p‐tau217 assay demonstrated excellent performance for detection of Aβ status in mild cognitive impairment/mild dementia. If used clinically in the current cohort, plasma p‐tau217 may have avoided more than half of diagnostic lumbar punctures.
3. **Future directions**: Real‐world replication studies are required in diverse populations. Such studies can provide an evidence base to support the development of precise pathways, cut points, and testing strategies for incorporating blood‐based markers such as p‐tau217 into diagnostic workflows.

The research‐use only (RUO) Lumipulse G pTau217 kit (REF: 81471/81472) was used to measure plasma p‐tau217. Supplied calibrators and standard curves were used to ensure optimal assay performance on a Lumipulse G600 II analyzer. Study samples were analyzed across 3 days. Six individual plasma samples were analyzed twice on two separate days to estimate inter‐assay coefficient of variation (CV). Three different sets of six plasma samples were tested in duplicate on each day to estimate intra‐assay CV. All other study samples were analyzed in singlicate.

### 2.3 Statistical analysis

Descriptive statistics are reported as means with standard deviations (SD)/medians with interquartile ranges (IQR)/proportions, with *t* tests/Mann–Whitney U tests being used to test for inter‐group comparisons. Normality testing used Shapiro–Wilk tests. Plasma p‐tau217 performance in detecting Aβ (positive CSF Aβ42:Aβ40)/T (positive CSF p‐tau181) status was assessed using receiver operating characteristic (ROC) and area under the curve (AUC) analysis with 95% confidence interval (95% CI). To compare AUCs, a DeLong test was used.

To explore potential cut‐offs to detect Aβ status, we used a two‐threshold approach,^18^ with 90%, 95%, and 97.5% sensitivities and specificities. Participants above specificity cut points were considered “high likelihood” of Aβ positivity, those below sensitivity cut points as “low likelihood” of Aβ positivity, and those between the upper and lower threshold as “intermediate likelihood” of Aβ positivity. For each range we report the positive predictive value (PPV) and the negative predictive value (NPV). Analyses used STATA v17.0 and GraphPad Prism v10.0.

## 3 RESULTS

### 3.1 Cohort description

Of 148 participants (69.4 ± 6.5 years; 54.1% female), 101 (68.2%) had MCI and 47 (31.8%) had mild dementia. Mean time from initial assessment to diagnosis adjudication was 120 ± 31 days. By CSF, 60.8% (90/148) were Aβ+. Of these, AD was adjudicated to be the suspected etiology in nearly all (95.8%; 86/90; Table 1). The four remaining Aβ+ individuals had a clinical presentation consistent with primary alpha‐synuclein pathology with co‐morbid amyloid pathology (Table 1). In Aβ– individuals, suspected etiology was Lewy body disease in eight, frontotemporal dementia in three, and vascular cognitive impairment in two. In the remainder negative for Aβ (*N* = 45), neurodegenerative etiology was not clear after diagnostic work‐up and consensus discussion—all of whom had MCI.

**TABLE 1:** Participant characteristics.

Characteristic	Aβ+ve (*n* = 90)	Aβ−ve (*n* = 58)
Age, years (mean; SD)	70.2 (6.4)	68.1 (6.4)
Sex, female (*n*; %)	53 (58.9%)	27 (46.6%)
Duration of symptoms, months (median; IQR)	18.5 (12–24)	18 (12–24)
Addenbrooke's Cognitive Assessment III (median; IQR)	68 (58–77)	77.5 (68–84)
Frontal assessment battery (median; IQR)	14 (11–16)	13 (11–16)
Clinical stage (Consensus diagnosis)		
MCI (*n*; %)	53 (58.9%)	48 (82.8%)
Mild dementia (*n*; %)	37 (41.1%)	10 (17.2%)
Primary pathology (consensus diagnosis)		
AD (*n*; %)	86 (95.6%)	0
Lewy body disease/dementia with Lewy bodies (*n*; %)	4 (4.4%)	8 (13.8%)
Frontotemporal dementia (*n*; %)	0	3 (5.2%)
Vascular cognitive impairment (*n*, %)	0	2 (3.4%)
Other—etiology unclear (*n*; %)	0	45 (77.6%)
Lumipulse G Plasma p‐tau217, pg/mL (median; IQR)	0.64 (0.35–0.93)	0.15 (0.13–0.23)

*Note*: One hundred forty‐eight individuals donated plasma samples to the Tallaght Institute of Memory and Cognition Biobank for Research in Aging and Neurodegeneration biorepository at time of diagnostic LP. Values are provided as means with SD or medians with IQRs and proportions as total number of individuals. Lumipulse G plasma p‐tau217 results are provided in pg/mL.

Abbreviations: Aβ, amyloid beta; AD, Alzheimer's disease; IQR, interquartile range; LP, lumbar puncture; MCI, mild cognitive impairment; SD, standard deviation.

### 3.2 Plasma p‐tau217 for the detection of Aβ status

**FIGURE 1:**
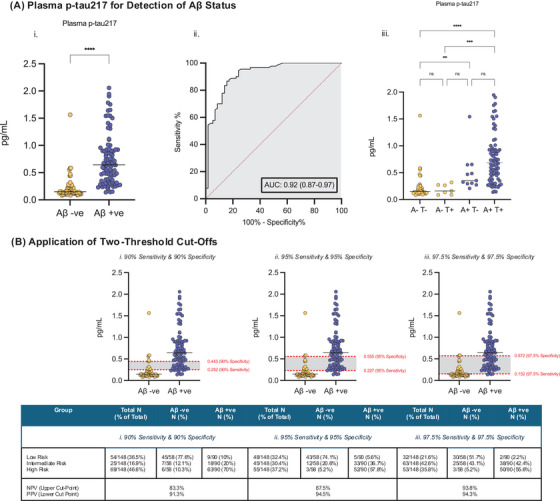
P‐tau217 performance for the detection of Aβ status in individuals undergoing diagnostic LP for early cognitive symptoms. A, (i) Plasma p‐tau217 by Aβ+ status, (ii) ROC for plasma p‐tau217 to detect Aβ status, (iii) plasma p‐tau217 by CSF‐defined A and T status. B, A two threshold approach with a lower cut‐off for sensitivity and a higher cut‐off for specificity at 90%, 95%, and 97.5% levels. ****P < 0.0001, ***P < 0.001, **P < 0.01, and *P < 0.05. A, amyloid; Aβ, amyloid beta; AUC, area‐under‐the‐curve; CSF, cerebrospinal fluid; LP, lumbar puncture; NPV, negative predictive value; ns, non‐significant; PPV, positive predictive value; p‐tau, phosphorylated tau; ROC, receiver operating characteristic; T, tau.

Plasma samples were analyzed using the Lumipulse p‐tau217 assay (intra‐assay CV: 8.3%; inter‐assay CV: 7.2%; lower limit of quantification: 0.03 pg/mL). Median plasma p‐tau217 level was > 4‐fold higher in Aβ+ (0.64 pg/mL; 0.35–0.93 pg/mL) versus Aβ− (0.15 pg/mL; 0.13–0.23 pg/mL) individuals (U = 426, *P* < 0.001; Figure 1). To detect Aβ positivity, plasma p‐tau217 had an AUC of 0.92 (95% CI: 0.87–0.97). For T pathology, plasma p‐tau217 had an AUC of 0.84 (95% CI: 0.78–0.91), significantly lower than that for Aβ (*z* = 2.04, *P* = 0.04 DeLong test).

### 3.3 Application of cut‐off thresholds for plasma p‐tau217

At 90% thresholds, 16.9% (25/148) of participants had p‐tau217 values in the intermediate range (Figure 1). Just under one third (30.4%; 45/148) at 95% thresholds and just over two fifths (42/6%; 63/148) at 97.5% thresholds were in the intermediate range.

At 90% thresholds, there were nine “false negatives” (p‐tau217 below sensitivity threshold yet Aβ+ on CSF) and six “false positives” (p‐tau217 above specificity threshold yet Aβ− on CSF). The false negatives at 90% sensitivity included two individuals with AD dementia, one with Lewy body disease and Aβ co‐pathology, and six with MCI due to AD. All six false positives at 90% specificity had MCI with unclear neurodegenerative etiology. All these individuals had normal renal function. At 95% thresholds, there were five false negatives/three false positives, and at 97.5% thresholds, there were two false negatives/three false positives. The NPV/PPV, respectively, were 83.3%/91.3% at 90% thresholds, 87.5%/94.5% at 95% thresholds, and 93.8%/94.3% at 97.5% thresholds.

Hypothetically, if only those with “intermediate likelihood” of Aβ pathology based on plasma p‐tau217 had undergone LP in the first instance, 57.4% to 83.1% of LPs could have been avoided (Figure 1).

## 4 DISCUSSION

In 148 individuals with MCI/mild dementia undergoing diagnostic workup in an RSMC, the fully automated Lumipulse assay demonstrated excellent performance for the detection of Aβ positivity. Lumipulse plasma p‐tau217 performance in our study is similar to previous studies,^11^ such as a large study in MCI/dementia in which Lumipulse plasma p‐tau217 demonstrated an AUC of 0.93 to detect Aβ status.^12^ Other recent studies have demonstrated comparable performance of Lumipulse and Simoa p‐tau217 assays.^13^, ^19^ Our study adds further evidence for excellent performance of the Lumipulse plasma p‐tau217 assay to detect Aβ status in MCI/mild dementia in a real‐world clinical cohort.

We applied a two‐threshold approach^12^, ^18^—to provide more stringent thresholds under which, in theory, only intermediate results would proceed to further testing.^12^ Recent guidelines report using a two‐threshold approach for clinical use; a BBM result should deliver an intermediate result in < 20% (at 90% sensitivity/90% specificity).^20^ In our data, at 90% sensitivity/90% specificity, intermediate results were observed in 16.9% of individuals.

Based on the implications of potential anti‐amyloid therapies and the impact of an AD diagnosis, aiming for thresholds with specificities > 90% may minimize false positives. In our data, the PPV of plasma p‐tau217 increased using 95%/97.5% thresholds resulting in fewer false positives. The NPV of plasma p‐tau217 increased from 83.3% (at 90% sensitivity) to 93.8% (at 97.5% sensitivity). Selection of higher sensitivity thresholds may result in fewer false negatives. The NPV may reflect reduced precision of plasma p‐tau217 at lower concentrations, and while plasma p‐tau217 exhibited excellent group‐level performance, careful clinical interpretation may be needed with results near the lower cut point when there is a strong index of suspicion for AD.

In conclusion, our data support the use of fully automated immunoassays for plasma p‐tau217 in individuals undergoing diagnostic workup in specialist memory services. Future larger studies should additionally consider the potential impact of renal function and vascular risk factors on plasma p‐tau217 performance in more diverse populations. Future studies should also incorporate the use of two‐threshold approaches as BBMs, such as p‐tau217, enter clinical use and exact clinical pathways based on plasma p‐tau217 results are defined, including optimal selection of cut points to minimize false negative and false positive results.

## AUTHOR CONTRIBUTIONS

Adam H. Dyer: conceptualization, methodology, formal analysis, investigation, data curation, writing – original draft, writing—review & editing, project administration; Jean Dunne: conceptualization, methodology, formal analysis, investigation, data curation, writing—original draft, writing—review & editing, project administration; Helena Dolphin: conceptualization, methodology, writing—review & editing, project administration; Laura Morrison: conceptualization, investigation, data curation, writing—review & editing, project administration; Antoinette O'Connor: conceptualization, investigation, data curation, writing—review & editing; Sarah Fullam: conceptualization, investigation, data curation, writing – review & editing; Tara Kenny: project administration; Aoife Fallon: investigation, data curation, writing—review & editing, project administration; Sean O'Dowd: conceptualization, investigation, data curation, writing—review & editing; Nollaig Bourke: conceptualization, investigation, supervision, writing—review & editing; Niall Conlon: conceptualization, investigation; Sean Kennelly: conceptualization, methodology, formal analysis, investigation, data curation, writing—original draft, writing—review & editing, project administration. TIMC‐BRAiN Collaborators: investigation, data curation, writing—review & editing.

## CONFLICT OF INTEREST STATEMENT

The authors declare no conflicts of interest. Author disclosures are available in the supporting information.

## CONSENT STATEMENT

All participants gave written informed consent to participate in the study

## ETHICAL APPROVAL

Full ethical approval for the TIMC‐BRAiN Study has been granted by the St. James's Hospital/Tallaght University Hospital Ethics Committee (Project ID: 2159), which operates in compliance with the European Communities Regulations 2004, ICH Good Clinical Practice Guidelines, and the Declaration of Helsinki.

## Supporting information



Supporting Information

## Data Availability

Due to the sensitive nature of participant data and the risk of identification, data are not publicly available; however, requests to the corresponding author (dyera@tcd.ie) will facilitate access to limited anonymized data.
